# Efficacy of Interventions to Promote Exercise Adherence in People With Overweight or Obesity: A Systematic Review

**DOI:** 10.1155/jobe/4164477

**Published:** 2026-01-09

**Authors:** Matheus de Sena Anchieta Rodrigues, Lívia de Melo Atanasio, Isis Kelly dos Santos, José Carlos Gomes da Silva, Breno Guilherme de Araujo Tinoco Cabral, Paulo Moreira Silva Dantas

**Affiliations:** ^1^ Department of Physical Education, Federal University of Rio Grande do Norte, Natal, Rio Grande do Norte, Brazil, ufrn.br; ^2^ Department of Physical Education, State University of Rio Grande do Norte, Mossoró, Rio Grande do Norte, Brazil, uern.br; ^3^ Graduate Program in Health Sciences, Federal University of Rio Grande do Norte, Natal, Rio Grande do Norte, Brazil, ufrn.br

**Keywords:** accession, adherence, obesity, overweight, physical exercises

## Abstract

**Background:**

The World Health Organization estimates that more than 500 million people will be affected by diseases related to physical inactivity in the next decade. Individuals with overweight or obesity are particularly vulnerable, making exercise adherence a critical public health concern. This review aimed to evaluate the efficacy of interventions designed to improve adherence to exercise in this population.

**Methods:**

A systematic search was conducted in MEDLINE, Embase, Virtual Health Library, Cochrane Library, and SPORTDiscus. Two independent researchers performed screening, data extraction, and synthesis of studies including adults aged 18–59 years with overweight or obesity. Eligible interventions lasted at least 12 weeks, included a control group, and reported adherence‐related outcomes. Methodological quality was assessed using the Cochrane Risk of Bias tool. When possible, meta‐analysis was performed.

**Results:**

Seventeen studies met the inclusion criteria. Group‐based programs and interventions supervised by trained professionals were consistently associated with higher adherence. Factors, such as body weight, exercise frequency, session duration, intensity, and type of intervention, showed no consistent influence. However, most studies presented a moderate to high risk of bias.

**Conclusion:**

Group and supervised interventions appear effective in improving exercise adherence among adults with overweight or obesity, but further high‐quality studies are needed.

## 1. Introduction

Physical activity in the early days of humanity was essential for survival; today, however, it has become a matter of choice. Humans now have the option of not being physically active; yet, this choice for inactivity negatively affects almost all body tissues, leading to disease and reduced longevity [1]. The World Health Organization (WHO) highlights that the economic development of countries has also had negative effects on population activity levels, making societies increasingly sedentary [2]. According to WHO projections (2020), in the next decade, sedentary behavior could lead to nearly half a billion people developing noncommunicable diseases, such as cardiovascular disease, diabetes, and obesity.

Despite such warnings, global adherence to physical activity remains low and was further compromised by the COVID‐19 pandemic, during which sedentary behavior and sitting time significantly increased [3]. Individuals with overweight or obesity are particularly vulnerable to this issue, as they often experience a feedback cycle in which physical inactivity contributes to weight gain, and increased body weight further reinforces inactivity [4].

According to WHO criteria, overweight is defined as a body mass index (BMI) ≥ 25 kg/m^2^, and obesity as BMI ≥ 30 kg/m^2^, conditions characterized by excessive body fat that can harm health [5]. This classification is not only clinical but also highlights the population at higher risk of facing barriers to sustained physical activity [6]. Indeed, obesity affected around 15% of the global population in 2010 and is projected to increase by 60% by 2030, reaching nearly one billion cases worldwide. Morbid obesity is expected to rise even more sharply, with estimates of over 100% growth in the same period [7].

These projections underscore the urgent demand for effective and scalable strategies to improve exercise adherence in this population, because to curb these trends requires more than simply initiating exercise; it demands the maintenance of this practice over extended periods, preferably lifelong, which previous reviews have highlighted as the main challenge for this population [6, 8].

Psychological and behavioral science theories provide valuable frameworks to understand this challenge: The self‐determination theory, for example, emphasizes autonomy, competence, and relatedness as drivers of intrinsic motivation, while the health belief model highlights perceived benefits, barriers, and self‐efficacy as key predictors of health behavior [8]. These perspectives are consistent with evidence showing that behavioral strategies, such as goal setting, self‐monitoring, and relapse prevention, can significantly improve adherence to lifestyle interventions in adults with obesity [6], and that determinants, such as social support and structured supervision, are particularly relevant in this population [8].

Previous reviews have provided valuable contributions but also revealed important gaps. Burgess et al. [6], for example, demonstrated that behavioral strategies improve adherence to lifestyle interventions, while Burgess et al. [8] identified social and contextual factors as major determinants of sustained participation. More recently, Wang et al. [9] synthesized 47 factors influencing adherence to weight‐loss interventions across psychological, behavioral, dietary, and pharmacological domains, but highlighted the heterogeneity of interventions and outcomes. Notably, these reviews did not isolate adherence outcomes specifically linked to exercise‐based interventions tested in randomized controlled trials (RCTs) of sufficient duration. This gap underscores the need for this systematic review.

Together, these projections and previous findings highlight the pandemic nature of obesity and physical inactivity across the globe, and confirm the importance of addressing these challenges as urgent public health priorities. However, effective intervention strategies to break this vicious cycle remain unclear. A systematic review of the literature can therefore provide critical insights into which interventions most effectively promote adherence to physical exercise in individuals with overweight or obesity. In addition, such evidence can support health promotion policies, inform clinical practice, and guide future research. Accordingly, the primary aim of this systematic review was to identify interventions that are effective in promoting exercise adherence in people with overweight or obesity, specifically by analyzing RCTs of at least 12 weeks in duration with a nonintervention control group, in adults aged 18–59 years. Studies with incomplete data or those involving hospitalized patients, individuals with disabilities, cancer patients, or pregnant or breastfeeding women were excluded.

## 2. Methods

This systematic review was conducted following the Preferred Reporting Items for Systematic Reviews and Meta‐analyses (PRISMA) reporting guide [10].

### 2.1. Search Strategy

An independent search was carried out without language restrictions in the following databases: MEDLINE, Embase, Virtual Health Library (BVS), Cochrane, and SPORTDiscus, by two independent researchers (MSAR and LMA). The complete search strategy is provided in Supporting Table S1. For the other databases, the search was adjusted according to their specific characteristics, noting that BVS, Cochrane, and SPORTDiscus do not support search filters by study type (Line 3), which was structured based on studies that demonstrated greater efficiency in retrieving RCTs [11].

### 2.2. Eligibility Criteria

**Figure 1:**
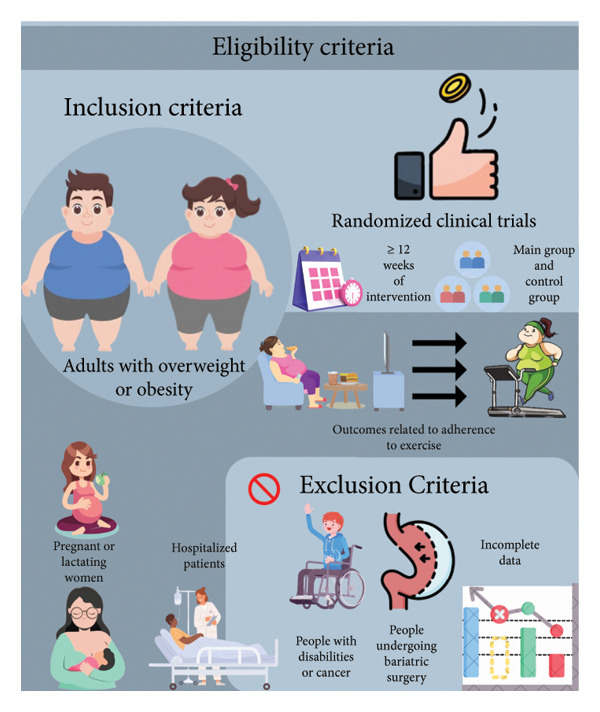
Eligibility criteria.

As illustrated in Figure 1, only RCTs with at least 12 weeks of intervention in the main group, a control group without the intervention, and outcomes related to adherence to physical exercise were included. The population comprised adults (aged 18–59 years) with overweight or obesity, defined as BMI ≥ 25 kg/m^2^. Interventions or outcomes not related to the focus of this review were not evaluated. Studies with hospitalized patients, pregnant or lactating women, people with disabilities or cancer, and those with incomplete data were excluded.

### 2.3. Data Recording

The studies selected to be part of this systematic review were evaluated independently by two researchers in two phases. First, titles and abstracts were screened to identify potentially relevant studies. Second, full‐text articles were assessed against the eligibility criteria. Disagreements were resolved by a third reviewer (IKS).

Data extraction was also performed independently and in duplicate by the two main evaluators, using the Rayyan systematic review manager (Rayyan Systems Inc., Cambridge, MA, USA; https://www.rayyan.ai/), which facilitated screening and data organization. As with study selection, disagreements were resolved by a third reviewer.

The extraction form collected the following information: general study characteristics (title, authors, year of publication, study design, and country of origin), participant characteristics (age, sex, and BMI), methodological aspects (objectives, randomization, and intervention and control protocols), and results (sample size, measurement tools, follow‐up duration, statistical tests, and descriptive data). In cases of missing data, attempts were made to contact study authors; when unsuccessful, only the published information was considered.

### 2.4. Risk of Bias Analysis of Included Studies

The risk of bias was assessed based on the analysis of the representativeness of the samples, the methods of selecting participants, measuring outcomes, and controlling confounding factors, in addition to potential conflicts of interest in the included studies. For this purpose, the Cochrane Risk of Bias tool Version 2.0 (RoB 2.0) was used. However, because this review focused specifically on adherence outcomes, an additional layer of assessment was necessary. Measures of adherence are often heterogeneous, ranging from attendance records to self‐reported participation, and these may introduce methodological challenges not fully captured by RoB 2.0.

To address this, seven independent criteria were applied, adapted from the QUADAS tool for diagnostic accuracy reviews [12], as previously used in similar contexts [13]. This adaptation allowed us to capture potential sources of bias specifically related to adherence reporting, such as the validity and reliability of measurement methods, the consistency of adherence definitions across groups, and the risk of bias introduced by incomplete or self‐reported data.

### 2.5. Data Synthesis

The evidence summary was presented as a narrative synthesis. Given the heterogeneity of the included studies—particularly regarding interventions—meta‐analysis was not performed. For topics with high heterogeneity, as in this case, narrative synthesis provides a more reliable integration of data, accounting for variability across study designs and methodologies [14]. This approach allowed us to assess the breadth of evidence linking adherence to exercise in adults with overweight or obesity while recognizing the diversity of factors and measures reported.

## 3. Results and Discussion

**Figure 2:**
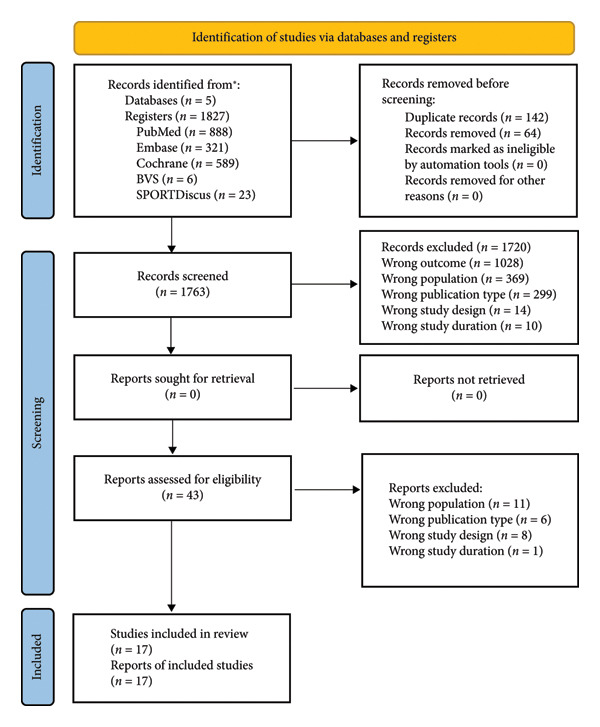
Study screening diagram.

The search in the five databases identified a total of 1827 studies, which, after a preliminary reading of the title and abstract, were reduced to 43 studies with the potential to meet all the criteria imposed for this review. The texts were analyzed in full to verify their fit with the objectives of this document, resulting in 17 articles that fully met the selection criteria, as shown in Figure 2.

A diagram of the study screening that shows the process of identifying eligible and ineligible articles, together with the criterion that excluded the latter, is as follows:

All selected studies were RCTs, according to the inclusion criteria, conducted mostly in Anglo‐Saxon America (United States: 8; Canada: 2), followed by European countries (Greece, Norway, Spain, and Switzerland: 1), Australia (2), and Iran (1), from 2007 to 2023.

Overweight and obesity are commonly linked to other chronic diseases. Although most studies do not present any information on this point, three of them included analyses with people with diabetes or prediabetes [15–17], two included analyses with people with dyslipidemia [15, 16], and samples with metabolic syndrome [18] and hypertension [15] were included in one study each.

The sample size varied greatly, from 15 to 947, with a median of 110. The majority of the population included people of both sexes (*n* = 10); six studies worked exclusively with females and only one study had exclusively males in its sample. The average age of the participants was 36–52.9, while the average BMI of the studies ranged from 28.6 to 47.8. It is not possible to specify an average or median for these topics, as Seif‐Barghi et al. [19] did not present information about them in their text, beyond the data reported in the eligibility criteria.

The intervention was the main point of heterogeneity among the studies, but in general, we can classify them into three large blocks, namely:

a. Physical Exercise: Contained in all selected interventions, with the exception of Lewis et al. [20] who brought purely psychological strategies. Being used in six studies as the only form of intervention;
b. Dietary Guidance: Addressed in six interventions, always accompanied by physical exercise and psychological strategies, with the exception of Batrakoulis et al. [21] who did not address psychological factors in their intervention;
c. Psychological Approaches: Also known in the literature as behavior change techniques (BCTs) [22]. This type of conduct was present in more than half of the studies (*n* = 9), always accompanied by exercise in their intervention, with the exception of the article by Lewis et al. [20] which, as mentioned above, used only psychological approaches related to commitment, planning, and goal achievement. It also combined nutritional guidelines in four studies [19, 23–25].

Another relevant aspect to mention is the use of technology in interventions, with emphasis on Spring et al. [26] and Apiñaniz et al. [15], who used mobile applications as the central point of their interventions.

All studies presented a control group, most of which had only this comparator (*n* = 11). Six articles included another comparator, in addition to the control group, which was an intervention different from the main one, usually addressing a shorter time interval or fewer intervention behaviors. Collins et al. [16] and Heiestad et al. [27] went further, including a control group and two other comparators, the first with behavioral decreases in relation to the main intervention and the last with interventions different from the main one, but which generated a similar internal load. Table 1 shows more information about the interventions and their comparators.

**Table 1:** Intervention summary and comparators.

Author, year	Intervention	Comparator 1	Comparator 2	Comparator 3
Batrakoulis et al., 2020	Hybrid training (aerobic resistance and strength) + nutritional monitoring	Hybrid training (aerobic resistance and strength) + 5‐month nutritional monitoring followed by 5‐month detraining	Control group	—
Annesi et al., 2011	Personalized training (details not reported) + cognitive‐behavioral protocol + nutritional information	Control group	—	—
Pettman et al., 2008	Aerobic and strength training + self‐monitoring of healthy behaviors + diet and exercise guidance	Control group	—	—
Spring et al., 2017	Behavior change strategies (psychologist) + aerobic training (walking) + mobile app	Behavior change strategies + aerobic training	Control group	—
Schmitz et al., 2007	Strength training guided by sociocognitive theory	Control group	—	—
Moss et al., 2017	Strength training + nutritional guidance + behavior change strategies + motivational interviewing	Control group	—	—
Seif‐Barghi et al., 2018	Aerobic exercise (walking) + cognitive‐behavioral therapy + low‐calorie diet	Control group	—	—
Lewis et al., 2019	Phone calls where people set goals for exercise and other healthy habits to achieve throughout the month	Control group (randomized crossover)	—	—
Williams et al., 2016	Walk	Control group	—	—
Schumacher et al., 2023	Exercise guidance (guided walk) to be performed in the morning	Guidance on exercises to be performed in the afternoon/evening	Control group	—
Collins et al., 2022	Aerobic exercise + resistance training + group behavioral counseling sessions	Aerobic exercise + resistance training	Aerobic exercise	Control group
Apiñaniz et al., 2019	Exercise and health guidance + mobile app	Control group	—	—
Heiestad et al., 2016	Bodypump	Personal trainer	Unsupervised exercise	Control group
Keshavarz; Sénéchal; Bouchard, 2023	Functional training	Control group	—	—
Annesi; Whitaker, 2008	Cognitive‐behavioral therapy + self‐selective affinity exercise + nutritional guidance + points related to dietary management	Cognitive‐behavioral therapy + self‐selective affinity exercise + nutritional guidance	Control group	—
Tudor‐Locke et al., 2014	Walking on a treadmill (at work)	The usual working group (control)	—	—
Berlin; Scholz, 2021	Exercise guidance + goal setting + text messaging + partner assistance (husband or wife)	Control group	—	—

Before presenting the outcomes, to assess the risk of bias, as defined in the methods, two resources were used, presenting the following results. The Cochrane risk of bias tool classified the studies into three possible statuses, according to the potential level of bias: low, moderate (some concerns), or high risk, based on the study assessment considering the five domains addressed by the tool, the studies were distributed as follows.

**Figure 3:**
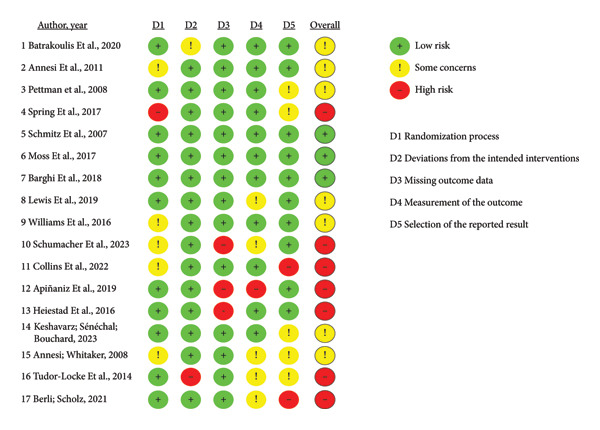
Risk of methodological bias.

In the first dimension, 11 studies were classified as low risk, five presented some concerns, and only the research by Spring et al. [26] was assessed as having a high risk of bias. In the second, only Batrakoulis et al. [21] and Tudor‐Locke et al. [17] had different classifications of low risk: some considerations and high risk, respectively. The third dimension had three studies that were classified as high risk [15, 27, 28], and the others had low risk. Arriving at the fourth dimension, the same distribution as in the first dimension was observed, but here the work evaluated as high risk was that of Apiñaniz et al. [15]. Finally, the last dimension classified 10 studies as having low bias, five with some concerns, and Berli and Scholz [29] and Collins et al.’s [16] articles with a high risk of bias. As a general result criterion, the tool adopts the worst classification of the five dimensions, and with this criterion, only 17.6% of the studies classified as low risk (*n* = 3) were perceived, with the other 82.4% divided equally between moderate (*n* = 7)‐ and high (*n* = 7)‐risk status. Figure 3 illustrates the assessment performed by the tool.

In addition to this tool, another tool was used that focused on the adherence data presented in each article, due to the relevance of this item for the review. This tool included seven questions that were answered with: (a) “Yes” (Y), if the study addressed that topic; (b) “No” (N), if the study did not address it; or (c) question mark (?), if the study did not provide information on the point. It had two studies responding positively [27, 30]. Tudor‐Locke [17] was the only one to respond negatively, and all the other authors did not address the issue in their research. This resulted in only two authors responding with “Yes” (Y) to all questions, which is the most favorable scenario possible [27, 30]. Most of them had six affirmative responses (11 or 64.7%), and four surveys had “Yes” (Y) reported in only five questions [16, 23, 25, 26]. Table 2 shows details of the responses from each study.

**Table 2:** Assessment of risk of bias linked to adherence.

Author, year	Q1^1^	Q2^2^	Q3^3^	Q4^4^	Q5^5^	Q6^6^	Q7^7^	Total/7
Batrakoulis et al., 2020	Y	Y	Y	Y	Y	?	Y	6
Annesi et al., 2011	Y	Y	Y	Y	N	?	Y	5
Pettman et al., 2008	Y	Y	Y	Y	Y	?	Y	6
Spring et al., 2017	Y	Y	Y	Y	N	?	Y	5
Schmitz et al., 2007	Y	Y	Y	Y	Y	Y	Y	7
Moss et al., 2017	Y	Y	Y	Y	N	?	Y	5
Seif‐Barghi et al., 2018	Y	Y	Y	Y	Y	?	Y	6
Lewis et al., 2019	Y	Y	Y	Y	Y	N	Y	6
Williams et al., 2016	Y	Y	Y	Y	Y	?	Y	6
Schumacher et al., 2023	Y	Y	Y	Y	Y	?	Y	6
Collins et al., 2022	Y	Y	Y	Y	N	?	Y	5
Apiñaniz et al., 2019	Y	Y	Y	Y	Y	?	Y	6
Heiestad et al., 2016	Y	Y	Y	Y	Y	Y	Y	7
Keshavarz; Sénéchal; Bouchard, 2023	Y	Y	Y	Y	Y	?	Y	6
Annesi; Whitaker, 2008	Y	Y	Y	Y	Y	?	Y	6
Tudor‐Locke et al., 2014	Y	Y	Y	Y	Y	?	Y	6
Berlin; Scholz, 2021	Y	Y	Y	Y	Y	?	Y	6
Total/17	17	17	17	17	13	2	17	

^1^Participant Characteristics: The article reported the clinical and demographic characteristics of the study population, including age, sex, concomitant treatments, comorbidities, and country in which the study was conducted.

^2^Method of Recording Adherence Data: The method used by participants to record or report adherence data to home exercises was clearly defined.

^3^Timing of Adherence Data Collection: The timing of collection of exercise adherence data by a study associate was clearly defined.

^4^Retrospective Recall of Adherence Period Data: There was no substantial time lag between completion of the exercise session and the recording or reporting of participant adherence. Adherence data were recorded or reported within 1 month of the time each exercise session was completed.

^5^Description of Intervention Components: The delivery of the intervention was described in sufficient detail to allow replication of the trial.

^6^Independent Verification of Adherence Data: All participant exercise adherence data were verified by an independent person, such as a supervisor or caregiver.

^7^Treatment of Indeterminate or Missing Adherence Data: The article reported whether or not there were missing or indeterminate adherence data and, if present, how the data were treated.

The interventions had a median of 24 weeks, ranging from 12 to 43 weeks of intervention. The study by Schmitz et al. [30] had the longest duration, 2 years. However, as occurred in six other articles, there was a shorter intervention time, and in the remainder of the process, we only observed the events that the intervention caused in the sample’s adherence to exercise.

Data on frequency, duration, or intensity of training sessions were not reported by several studies included in this review, with six studies without information on the first two items, five for the third and just nine of them expressly reporting the three markers [16, 18, 19, 21, 27–29, 31, 32].

The weekly frequency of interventions varied from at least one to seven days, and the session duration was at least 15–60 min, making it not possible to express the mean or median precision of the two variables, as in some studies, the authors reported intervals as sessions of 30–60 min, for example. The intensity of the training sessions was primarily moderate to vigorous. Three authors used only moderate exercise intensities. Only Batrakoulis et al. [21] worked at exclusively vigorous intensities and Tudor‐Locke et al. [17] worked exclusively with light intensity.

Most interventions occurred in a self‐monitored manner (*n* = 10). Participants received guidance from researchers/instructors and put them into practice without on‐site supervision from professionals, reporting compliance with the intervention later through paper reports or using technologies, such as pedometers or cell phone applications. Four studies took place with treatment sessions supervised by researchers/instructors, and three of them took place in a mixed format, with moments of supervision and moments of self‐monitoring.

In six studies, these treatment sessions took place in groups. Nine were completed individually by the selected participants and two occurred in a mixed way, with moments of group activity and individual moments. More information about the interventions is shown in Table 3.

**Table 3:** Intervention data.

Author, year	Intervention time (weeks)	Weekly frequency	Session duration (min)	Intensity	Type of follow‐up	Group or individual treatment
Batrakoulis et al., 2020	43	3	38–41	Vigorous	Instructor supervised	Group
Annesi et al., 2011	26	Not reported	Not reported	Not reported	Self‐monitored	Individual
Pettman et al., 2008	52 (16^∗^)	≥ 1	45–60	Moderate/vigorous	Mixed	Mixed
Spring et al., 2017	52 (26^∗^)	Not reported	Not reported	Moderate/vigorous	Mixed	Mixed
Schmitz et al., 2007	104 (16^∗^)	2	45–60	Not reported	Instructor supervised	Group
Moss et al., 2017	26 (12^∗^)	Not reported	Not reported	Not reported	Self‐monitored	Group
Seif‐Barghi et al., 2018	16	7	50	Moderate	Self‐monitored	Individual
Lewis et al., 2019	34 (17^∗^)	Not reported	Not reported	Not reported	Self‐monitored	Individual
Williams et al., 2016	26	5	30–60	Moderate	Self‐monitored	Individual
Schumacher et al., 2023	13	7	≥ 15	Moderate	Self‐monitored	Individual
Collins et al., 2022	26–34	≥ 2	≤ 60	Moderate/vigorous	Self‐monitored	Individual
Apiñaniz et al., 2019	26	Incomplete information	Not reported	Moderate/vigorous	Self‐monitored	Individual
Heiestad et al., 2016	12	3	45–60	Moderate/vigorous	Instructor supervised	Group
Keshavarz; Sénéchal; Bouchard, 2023	46 (12^∗^)	3	50	Moderate/vigorous	Mixed	Group
Annesi; Whitaker, 2008	24	3	Not reported	Not reported	Self‐monitored	Group
Tudor‐Locke et al., 2014	26	Not reported	45	Light	Instructor supervised	Individual
Berlin; Scholz, 2021	26	7	≥ 30	Moderate/vigorous	Self‐monitored	Individual

^∗^Weeks of effective intervention, and the rest was monitoring the effects generated by this intervention.

Many variables were assessed in the study outcomes, but for the purposes of this review, only adherence to physical exercise was considered. As a criterion for assessing adherence, most studies used the number of sessions/days/minutes actually attended, divided by the total number of sessions/days/minutes prescribed (*n* = 14). In addition to this approach, Seif‐Barghi et al. [19] and Lewis et al. [20] used the change in the number of steps pre‐ and postintervention to assess adherence, as did Williams et al. [32], who made this measurement through accelerometer data, combining the average duration of walking sessions with the interval of days between consecutive sessions, going beyond simple attendance records.

The intervention groups had adherence levels ranging from 24% to 93.5%, in the studies that used percentage as a way of expressing the data (*n* = 14), with an average of 64% among them. The control groups in these same studies had an average of 34%, with raw data ranging from 5.3% to 83%, although it is important to note that seven studies did not report the adherence of this group.

The total number of sessions and minutes of treatment varied considerably between the articles, ranging from 14 to 130 sessions and from 630 to 7800 min during the entire intervention, with averages of 75.4 sessions and 2520.5–3313.5 min per intervention. The average number of minutes varied between a maximum and a minimum, as some studies included intervals per minute, such as 30–60 min. Therefore, we can conclude that the average session time in the studies eligible for this review was close to 40 min. It is important to note that six studies did not provide information on the number or duration of sessions in their experiment.

The samples managed to complete, on average, 37.3 sessions, participating for an average of 1210.8–1316.1 min in each intervention. This adherence ranged from 9 to 136 treatment sessions, with 405–7820 min completed. Based on these values, we can infer that adherence to the treatment sessions that had their numbers reported was close to 50%, with participants completing 39.7, 48% of the planned minutes in the studies that reported these data. Ten studies did not present data on the topics covered in this paragraph. More details on adherence data are shown in Table 4.

**Table 4:** Adhesion data.

Author, year	Level of adherence of the intervention group	Level of adherence of the control group	Assessment tools
Batrakoulis et al., 2020	93.5% ± 2.0	Not rated	The number of sessions actually attended, divided by the total number of sessions offered
Annesi et al., 2011	49.27% ± 28.89	31.07% ± 25.04	The proportion of sessions attended divided by the number of sessions offered, expressed as a percentage. Completed exercise sessions were recorded electronically via a computer system.
Pettman et al., 2008	66%^∗^	Not rated	The number of sessions actually attended, divided by the total number of sessions offered. Assessed through attendance records and weekly exercise records
Spring et al., 2017	56.8% ± 4.8	9.8% ± 2.4	The number of sessions actually attended, divided by the total number of sessions offered. Assessed through the frequency reported in the paper diary (STND and SELF) or when some physical activity was detected on the accelerometer (TECH).
Schmitz et al., 2007	71%^∗^	Not rated	The number of sessions actually attended, divided by the total number of sessions offered. Assessed based on participants’ exercise records maintained at the YWCA and checked weekly by fitness instructors.
Moss et al., 2017	84.5% ± 82.9	83% ± 85	The number of sessions actually attended, divided by the total number of sessions offered
Seif‐Barghi et al., 2018	3195.53 ± 5428.39^∗∗^	−1213.95 ± 5311.48^∗∗^	Days with 5000 steps completed, with a frequency of at least 100 steps per minute or 3 METs, monitored by pedometer
Lewis et al., 2019	658^∗∗^ [−176; 1494]^∗∗∗^	1151^∗∗^ [353; 1949]^∗∗∗^	Change in number of steps using activity monitors worn for seven consecutive days. The SenseWear Pro3 Mini Armband was the chosen monitor.
Williams et al., 2016	9.43 ± 2.88^∗∗∗∗^	12.50 ± 3.42^∗∗∗∗^	Ratio between exercise duration and latency.
Schumacher et al., 2023	83.8% ± 27.7	Not rated	The number of sessions actually attended, divided by the total number of sessions offered
Collins et al., 2022	85.1% ± 16.2	Not rated	Aerobic Training: Weekly minutes of exercise completed after the ramp period divided by the weekly minutes of exercise prescribed after the ramp period. Resistance Training: Total weekly sets completed divided by the total weekly sets prescribed after the ramp period.
Apiñaniz et al., 2019	75% (59–91)^∗∗∗^	56% (36.5–75.5)^∗∗∗^	The number of sessions actually attended, divided by the total number of sessions offered
Heiestad et al., 2016	72.5% ± 28.6	Not rated	The number of sessions actually attended (entries in the exercise diary), divided by the total number of recommended training sessions
Keshavarz; Sénéchal; Bouchard, 2023	36.8%^∗^	5.3%^∗^	Number of weeks with 150 minutes of moderate‐to‐vigorous intensity aerobic activity monitored by validated Fitbit Charge 3 and reporting at least two sessions of muscle‐strengthening activities, divided by the total number of weeks in the study
Annesi; Whitaker, 2008	50.97% ± 30.03	31.07% ± 25.04	The proportion of sessions attended divided by the total number of sessions offered, expressed as a percentage
Tudor‐Locke et al., 2014	∼50%^∗^	Not rated	Percentage of treadmill sessions attended, divided by the total number of recommended training sessions
Berlin; Scholz, 2021	24% ± 25	22% ± 22	Days with 30 or more minutes of physical exercise performed in sessions of at least 10 minutes were coded as 1 (adherent days), and days with less than 30 minutes were coded as 0 (nonadherent days).

^∗^Standard deviation not mentioned.

^∗∗^Based on the difference in steps between the 1st and 16th weeks.

^∗∗∗^Standard deviation not reported, only the variation between the data.

^∗∗∗∗^Based on the relationship between exercise duration and latency.

The objective of this review was to identify which interventions are effective in promoting adherence to physical exercise in people with overweight or obesity. In this sense, 17 studies were identified that could satisfy this requirement, respecting the eligibility criteria. However, just over half of the studies analyzed had adherence as the main focus of the intervention [16, 17, 19–21, 23, 31, 32]. The remaining articles focused mainly on changes in body composition. This made these findings more limited, as when planning the interventions, the primary researchers did not target or seek strategies that could potentially lead to a more positive outcome with regard to adherence to exercise.

Another critical point found was the perception of relatively low quality of the articles that met the eligibility criteria, as shown in Figure 3. Of the 17 articles analyzed, only one presented a low risk of bias and responded positively to all questions assessing biases related to adherence [30]. This means that the findings, although mostly positive, cannot be fully interpreted as true, making it impossible to answer exactly which interventions are effective for promoting adherence to physical exercise in people with overweight or obesity.

The other specific aims of this analysis were achieved by identifying the interventions already tested in RCTs for promoting adherence to exercise in people with overweight or obesity, finding great heterogeneity among the interventions. It is interesting to note that half of the articles using only exercise as a strategy to generate greater adherence are on the list of studies that had adherence as the primary focus of the research. This is interesting, as the WHO itself recommended, well before the publication of these studies, that the strategy for promoting adherence to exercise in people with chronic diseases, such as those affected by obesity, should involve multiple factors, such as health education and behavioral tools [33].

Analyzing the interventions tested, it was observed that adherence to exercise was positive in most cases. As presented by Schumacher et al. [28], interventions that promote adherence equal to or greater than 60% can be considered successful. The highlight in this regard was the research by Batrakoulis et al. [21], which achieved an average of 93.5% adherence in the 10 months of their hybrid training (aerobic and strength training), supervised by a trained instructor. However, in this research, people who missed more than 20% of the exercise sessions offered were excluded from the study, a fact that may have biased the data. The aforementioned study had a dropout rate of 22.4%, with no specific reason for each exclusion being mentioned. Next to it, the study with the best adherence percentage was that of Collins et al. [16], which also used a hybrid training strategy, with aerobic and strength exercises combined with group behavioral counseling sessions and achieved an adherence rate of 85.1%.

With an average below the ideal of 60%, we had six studies, as shown in Table 3, with a negative highlight for the study by Berli and Scholz [29], which worked with exercise guidance (performed in an unsupervised manner), goal setting, and motivational text messages, or with behavioral guidelines and assistance from the partner (husband or wife). This study achieved the lowest percentage among the studies that reported adherence with this metric, at just 24%, only 2% more than the adherence reported for the control group, which differed from the intervention group by not establishing exercise goals.

However, even though it did not generate a high level of adherence and did not generate significant changes in relation to the control, this research showed an interesting fact: 20% of the partners who were helping their spouse in the intervention adhered to the exercise guidelines provided in the research, which may be a good start to generate a change in behavior in people who are in a state of contemplation and even precontemplation. Logically, robust and well‐designed studies should be carried out to confirm this hypothesis, but this may be an excellent start, as 85.7% of adults with obesity do not exercise as recommended, of at least 150 min at moderate intensity or 75 at vigorous intensity, which was also the goal in the study in question [34].

In the studies that showed lower adherence, we have Keshavarz et al. [31] with 36.8%, who exposed their population to “traditional” functional training, carried out three times a week, for 50 min, one accompanied by a trained instructor and the other self‐monitored. One point that may have contributed to this lower‐than‐expected number is that the intervention, in addition to not using other approaches besides exercise, took place for 12 weeks and the study continued to monitor this population for almost 8 months after that, a fact that may have harmed the research, when compared to others that carried out the intervention throughout the study period. Although two other studies had the same characteristic (intervention time followed by 8 months or more of observation), obtained results were above 60% adherence.

Despite the heterogeneity already mentioned, we can see some similarities between the articles that obtained the best adherence levels. For example, 83.3% of the studies that carried out group interventions exceeded adherence expectations, while this number in interventions carried out individually was only 50%. Two interventions were mixed, with one part carried out in groups and the other part carried out individually, and both were close to the 60% adherence target, but one was above and the other below. All of these were for the studies that reported adherence in the form of a percentage.

The same behavior was observed when comparing studies that had their interventions supervised by instructors and those that were carried out in a self‐monitored or mixed manner (mostly self‐monitored, but supervised by instructors at specific times). In the former, satisfactory adherence levels were observed in 75% of cases, although it should be emphasized that only four studies opted for this form of monitoring. In the latter, there was a larger volume, 10 articles in total, where a balance was observed between failure and success of the interventions regarding adherence to exercise, with five of them being above the 60% set here as the cutoff point and the other five being below.

These data lead us to believe that social interaction is an important weapon when thinking about engagement in physical exercise in people with overweight or obesity. Even more so, when we observe that in all the articles the intervention was carried out in groups, and with professional supervision, they obtained high levels of adherence (71% and 93.5%). However, only two papers brought this characteristic and, in addition, one of these studies presented some concerns about the risk of bias in its intervention.

Taking BMI as a reference, no major impact of this variable was observed on adherence results, as the interventions that met and did not meet expectations had similar BMI averages: 33.4 and 33.9, respectively. Within the BMI ranges, this was also repeated with the studies with an average of overweight, with three studies, two above and one below the average; the five studies on grade I obesity with three above and two below expectations; and the six studies on grade II obesity with half exceeding the target. Another piece of data that corroborates the hypothesis that BMI does not have a major impact on adherence to exercise is that the two studies that obtained the best and worst results in this outcome had populations with similar averages. Likewise, consider the articles that reported adherence as a percentage, as will be in the following paragraph.

Regarding the intervention, it was also not clear which approaches generated higher levels of adherence. Logical thinking, based on the WHO [33] recommendation, cited a few paragraphs above, would lead us to believe that strategies with multiple factors could have better results. However, the studies analyzed showed that the articles that worked with psychological aspects or supported by technology had similar percentages of success and failure in reaching the 60% adherence target set as the cutoff point, 35.7% and 28.8%, respectively, with five and four articles as absolute numbers. Likewise, the studies that used only exercise as a strategy obtained an equal rate of success and failure, 14.2%, with two studies on each side.

However, the time of the intervention, in these same articles, brought a much more defined data. With the caveat that there were only two studies in this condition, none of the interventions with 3 months showed adherence below the established cutoff point, while the interventions with 6 months showed 57.1% of the studies falling short of this goal, and those with a time of more than 6 months, 40%. Thus, there does not seem to be any differentiation in the period of exposure within this time; as of the five studies that had an effective intervention time and then an additional observation time, three had adherence greater than the expected 60% and two did not meet this expectation.

This fact leads us to believe that, regardless of the chosen intervention strategy, it will go well until the third month, and greater attention should be paid by the professional, researcher, or patient from this moment until the sixth month. However, it was not possible to specify how this attention should be given and what should be done in it, as pointed out in the paragraph above, also leaving this question open for future studies. Other important data on the intervention, however, left a low level of evidence because they were not reported in several studies, such as weekly frequency, session duration, and exercise intensity, which were not reported in approximately 30% of the studies.

In terms of concrete data, in the studies that reported adherence in percentage, we have seven studies with a frequency of up to three times a week, with only two of them falling below expectations regarding adherence, and two studies with a frequency of seven times, with one of them falling short of the predefined goal. This could indicate, if there were more data on this item, that weekly frequency also does not make people with overweight or obesity adhere, more or less, to physical exercise.

Regarding session duration, there were three articles that suggested the possibility of the sample exercising for a duration of up to 30 min, two above and one below the goal. In those that required durations above this number, an average of two‐thirds of the studies also reached the desired 60% adherence. This could lead to the conclusion that training sessions lasting 15–60 min generate the same level of engagement in people with overweight or obesity. However, as previously mentioned, the high risk of bias in the studies and the low number of studies reporting these data make this idea unclear.

The intensity of the exercise presented seven interventions ranging from moderate to vigorous exercise, with a very similar percentage of success and failure among them. When taking into account adherence to the exercise, four reached the 60% cutoff point and three failed to reach this level. With intensities being required exclusively in the same range, we had one study with each level of intensity: light, moderate, and vigorous. Of these three, only the study with light intensity did not achieve the expected adherence.

Regarding the studies that did not assess adherence in percentage, there were two that used the number of steps [19, 20] and one that used the latency between workouts [32] for this purpose. Seif‐Barghi et al. [19] were one of the few authors who presented a low risk of bias, which makes their findings more relevant than most. They worked with people with an average BMI, close to grade II obesity, using nutritional guidance, psychological interventions, and physical exercises as a strategy, part of which was carried out in groups and the other part individually, without the presence of an instructor. The intervention lasted 16 weeks, with training sessions taking place every day, at moderate intensity for 50 min. The conclusion was a favorable outcome for adherence to exercise in the population tested.

Lewis et al. [20] used a sample of people with an average BMI slightly higher than 35, and their experiment lasted 34 weeks, 17 of which were intervention weeks and the rest were observation weeks. In their methodology, they used telephone calls so that instructors could help the population set and meet their individual goals for physical activity and other healthy habits. For this reason, the research did not report weekly frequency, session duration, or exercise intensity, as each participant was free to choose all of these variables, depending on their goals.

The conclusion that the researchers reached is that this is a positive strategy and can be incorporated into community obesity management services without much cost, given that the study presented a reduced risk of bias that gives rise to some concerns. Williams et al. [32] did not present satisfactory results for the outcome of adherence to physical exercise. However, there are reservations about this conclusion, as their research presented a risk of bias in the same classification as Lewis et al. [20]. To achieve this goal, he carried out an intervention using only physical exercise, performed in groups, but without the supervision of an instructor. The exercise was performed at a moderate intensity, for 30–60 min, five times a week, for a period of 26 weeks.

In view of this scenario, this review found the following gaps that should be filled in future studies: What interventions, or combinations of them, lead to better adherence to physical exercise by people with overweight or obesity? What is the ideal intensity, weekly frequency, and duration of the physical exercise session to achieve the aforementioned goal? And what actions should be taken after 6 months of intervention to maintain the adherence observed in the first 3 months? In addition to elucidating what happens to this adherence in this gap between the third and sixth months, at what point does it begin to decline more significantly?

Furthermore, some aspects were not observed in this review and may lead to some divergence in the answer to the question of which interventions successfully promote adherence to physical exercise in people with overweight or obesity, such as sex, age group, marital status, and other comorbidities affecting the individual with overweight or obesity. As men and women have very different routines, people aged 18 tend to have different priorities than individuals aged 50, for example. Married and single people have very different obligations and commitments, and people with overweight or obesity with other comorbidities have a series of limitations that can make adherence to exercise a much more complicated task.

Although every effort was made to conduct this review with excellence, some limitations must be acknowledged, such as the fact that some relevant studies may have been missed because they were only in the gray literature, in databases that were not searched, or simply because they were not sensitive to the search strategy chosen. The possibilities for intervention were completely open. Like any strategy, regardless of the field of knowledge, that aimed to promote adherence to physical exercise in people with overweight or obesity, it was included in the review, which generated great heterogeneity in the interventions, even making meta‐analysis of the data unfeasible. A possible publication bias in the studies led by Annesi and Whitaker in 2008 [24] and 2011 [23], which appear to have results published in duplicate, could generate overestimated data [35].

That said, it is strongly recommended to invest in research, preferably RCTs with at least 12 weeks of intervention and a control group. That work on the issue of adherence in people with overweight or obesity with greater methodological rigor, with attention to minimizing the risk of bias, analysis of adherence was carried out every month, with periodic assessments to measure other important aspects in combating sedentary lifestyle and excess weight with level of physical activity, BMI, or body composition.

The results of these studies should be adjusted, if possible, by BMI classification, age group, and associated comorbidities, among other factors that may influence adherence to exercise. The method also includes group work and training with qualified instructors to assess whether these social interactions are really decisive for greater adherence to physical exercise in the population studied. In this way, in the future, with an increase in the number of studies on this topic, new reviews can be carried out to further clarify the topic in question.

## 4. Conclusion

Although the studies presented important findings to elucidate the main question of this work, group activities or those monitored by a qualified instructor generate greater adherence to physical exercise in people with overweight or obesity. Regardless of the chosen intervention, this adherence will be satisfactory in the first 3 months, or even indicate factors that do not seem to affect adherence, such as BMI, weekly frequency, duration of the session, and intensity or type of intervention (only exercise or combinations with other strategies such as psychology).

The vast majority of the studies evaluated presented low methodological quality and the lack of mention of a series of important information. This fact leads to the conclusion that the findings described above should not be interpreted as reliable and that more robust studies with greater methodological rigor should be carried out based on these findings to answer the question with greater quality.

**Nomenclature**
WHO: World Health Organization
COVID‐19: Coronavirus disease 2019
BMI: Body mass index
kg/m^2^
: Kilograms per square meter
RCTs: Randomized clinical trials
PRISMA: Preferred Reporting Items for Systematic Reviews and Meta‐Analyses
MEDLINE: Medical Literature Analysis and Retrieval System Online
Embase: Excerpta Medica Base
BVS: Virtual Health Library
QUADAS: Quality Assessment of Diagnostic Accuracy Studies
BCTs: Behavior change techniques

## Ethics Statement

The original protocol was prospectively registered (CRD42023398679) in the International Prospective Register of Systematic Reviews (PROSPERO).

## Conflicts of Interest

The authors declare no conflicts of interest.

## Author Contributions

Matheus de Sena Anchieta Rodrigues: data acquisition, analysis and interpretation, and essay writing.

Lívia de Melo Atanasio: data acquisition and analysis and interpretation.

Isis Kelly dos Santos: data acquisition.

José Carlos Gomes da Silva: adaptation of the original dissertation into article format and assistance in updating and organizing references.

Breno Guilherme de Araujo Tinoco Cabral: cosupervision of the study, and contribution to conception, study design, and critical revision of the manuscript.

Paulo Moreira Silva Dantas: conception, design, and review of the work.

## Funding

This study is self‐funded.

## Supporting Information

The following supporting information is available with this article:

Table S1: Search strategy: Complete search strategy used MEDLINE/PubMed.

## Supporting information


**Supporting Information** Additional supporting information can be found online in the Supporting Information section.
