# Supplementary material for: Efficacy of Interventions to Promote Exercise Adherence in People With Overweight or Obesity: A Systematic Review
Source: J Obes. 2026 Jan 9;2025:4164477. doi: 10.1155/jobe/4164477 (PMC12784378; doi:10.1155/jobe/4164477)
Supplement: Supplementary file 1 — Supporting Information Additional supporting information can be found online in the Supporting Information section. [file JOBE-2025-4164477-s001.docx]

| **Search strategy for MEDLINE/PubMed** | | |
| --- | --- | --- |
| #1 | Population | ("Obesity"[Mesh] OR (obes*)) NOT ("Child"[Mesh] OR (Children) OR "Adolescent"[Mesh] OR (Adolescents) OR (Adolescence) OR (Teens) OR (Teen) OR (Teenagers) OR (Teenager) OR (Youth) OR (Youths) OR (Adolescents, Female) OR (Adolescent, Female) OR (Female Adolescent) OR (Female Adolescents) OR (Adolescents, Male) OR (Adolescent, Male) OR (Male Adolescent) OR (Male Adolescents) OR "Aged"[Mesh] OR (Elderly)) |
| #2 | Intervention | "Guideline Adherence"[Mesh] OR (Adherence) OR (exercise adherence) OR (non-adherence) OR (compliance) OR (non-compliance) AND ("Exercise"[Mesh] OR (Exercises) OR (Physical Activity) OR (Activities, Physical) OR (Activity, Physical) OR (Physical Activities) OR (Exercise, Physical) OR (Exercises, Physical) OR (Physical Exercise) OR (Physical Exercises) OR (Acute Exercise) OR (Acute Exercises) OR (Exercise, Acute) OR (Exercises, Acute) OR (Exercise, Isometric) OR (Exercises, Isometric) OR (Isometric Exercises) OR (Isometric Exercise) OR (Exercise, Aerobic) OR (Aerobic Exercise) OR (Aerobic Exercises) OR (Exercises, Aerobic) OR (Exercise Training) OR (Exercise Trainings) OR (Training, Exercise) OR (Trainings, Exercise) OR "Healthy Lifestyle"[Mesh] OR (Lifestyle, Healthy) OR (Lifestyles, Healthy) OR (Healthy Life Styles) OR (Healthy Lifestyles) OR (Healthy Life Style) OR (Life Style, Healthy) OR (Life Styles, Healthy)) AND (intervention) |
| #3 | Type of Study | (randomized controlled trial [pt] OR controlled clinical trial [pt] OR randomized controlled trials [mh] OR random allocation [mh] OR double-blind method [mh] OR single-blind method [mh] OR clinical trial [pt] OR clinical trials [mh] OR (“clinical trial” [tw]) OR ((singl* [tw] OR double* [tw] OR trebl* [tw] OR tripl* [ tw]) AND (mask* [tw] OR blind* [tw])) OR (“latin square” [tw]) OR placebos [mh] OR placebo* [tw] OR random* [tw] OR research design [mh] :noexp] OR comparative study [mh] OR evaluation studies [mh] OR follow-up studies [mh] OR prospective studies [mh] OR cross-over studies [mh] OR control* [tw] OR prospective* [tw] OR volunteer* [tw]) NOT (animal [mh] NOT human [mh]) |
| #4 |  | #1 AND #2 AND #3 |

**Table S1 - Search strategy**

The search strategy was adapted for each database, considering their specific characteristics (e.g., BVS, Cochrane, and SPORTDiscus do not support study type filters as in Line 3).
